# NAVIGATOR: a phase 3 multicentre, randomized, double-blind, placebo-controlled, parallel-group trial to evaluate the efficacy and safety of tezepelumab in adults and adolescents with severe, uncontrolled asthma

**DOI:** 10.1186/s12931-020-01526-6

**Published:** 2020-10-13

**Authors:** Andrew Menzies-Gow, Gene Colice, Janet M. Griffiths, Gun Almqvist, Sandhia Ponnarambil, Primal Kaur, Gennaro Ruberto, Karin Bowen, Åsa Hellqvist, May Mo, Esther Garcia Gil

**Affiliations:** 1 Royal Brompton Hospital, Sydney Street, London, SW3 6NP UK; 2 Late Respiratory & Immunology, BioPharmaceuticals R&D, AstraZeneca, Gaithersburg, MD USA; 3 Translational Science and Experimental Medicine, Early Respiratory & Immunology, BioPharmaceuticals R&D, AstraZeneca, Gaithersburg, MD USA; 4 Late Respiratory & Immunology, BioPharmaceuticals R&D, AstraZeneca, Gothenburg, Sweden; 5 Late Respiratory & Immunology, BioPharmaceuticals R&D, AstraZeneca, Cambridge, UK; 6 Amgen, Thousand Oaks, CA USA; 7 BioPharma Study Management, Late Respiratory & Immunology, BioPharmaceuticals R&D, AstraZeneca, Mississauga, ON Canada; 8 Biometrics, Late Respiratory & Immunology, BioPharmaceuticals R&D, AstraZeneca, Gaithersburg, MD USA; 9 Biometrics, Late Respiratory & Immunology, BioPharmaceuticals R&D, AstraZeneca, Gothenburg, Sweden; 10 Global Medical Respiratory, BioPharmaceuticals Medical, AstraZeneca, Barcelona, Spain

**Keywords:** Clinical trial, Severe asthma, Tezepelumab, Thymic stromal lymphopoietin

## Abstract

**Background:**

Patients with severe, uncontrolled asthma have a significant unmet need for new treatments that have broader effects on airway inflammation, and that provide greater improvements in asthma outcomes than currently approved biologics and standard-of-care therapies. Tezepelumab is a human monoclonal antibody that blocks the activity of the epithelial cytokine thymic stromal lymphopoietin. In the PATHWAY phase 2b study (NCT02054130), tezepelumab significantly reduced exacerbations by up to 71% in adults with severe, uncontrolled asthma, irrespective of baseline disease phenotype. This article reports the design and objectives of the pivotal phase 3 NAVIGATOR study.

**Methods:**

NAVIGATOR (NCT03347279) is an ongoing randomized, double-blind, placebo-controlled trial in adults (18–80 years old) and adolescents (12–17 years old) with severe, uncontrolled asthma, who are receiving treatment with medium- or high-dose inhaled corticosteroids plus at least one additional controller medication with or without oral corticosteroids (*N* = 1061). The study population includes approximately equal proportions of patients with high (≥ 300 cells/μL) and low (< 300 cells/μL) blood eosinophil counts. The study comprises a 5–6-week screening period, a 52-week treatment period and a 12-week post-treatment follow-up period. All patients will receive their prescribed controller medications without change throughout the study. The primary efficacy endpoint is the annualized asthma exacerbation rate during the 52-week treatment period. Key secondary endpoints include the effect of tezepelumab on lung function, asthma control and health-related quality of life.

**Discussion:**

NAVIGATOR is evaluating the effect of tezepelumab in patients with a broad range of severe asthma phenotypes at baseline, including those with low blood eosinophil counts. The target sample size for NAVIGATOR (*N* = 1060) was achieved, and it is the largest clinical study of tezepelumab in severe, uncontrolled asthma to date. NAVIGATOR aims to further investigate the effect of tezepelumab on exacerbations and build on observations from the phase 2b PATHWAY study, and to demonstrate further the potential of tezepelumab to provide patients with severe, uncontrolled asthma with improvements in lung function, asthma control and health-related quality of life.

**Trial registration:**

NCT03347279 (ClinicalTrials.gov). Registered 20 November 2017.

## Background

Asthma is a chronic inflammatory disease of the airways that occurs in approximately 339 million individuals globally [1], up to 10% of whom have severe asthma [2]. Many patients with severe asthma continue to experience asthma symptoms and exacerbations despite treatment with medium- to high-dose inhaled corticosteroids (ICS) and long-acting β_2_ agonists (LABAs), plus additional controller therapies [3]. These patients are at a high risk of exacerbations and experience twice as many asthma-related hospitalizations as those with non-severe disease [4]. Furthermore, severe, uncontrolled asthma is associated with high healthcare costs [5] and poor health-related quality of life (HRQoL), owing to excessive symptoms, frequent and life-threatening exacerbations, increased comorbidity burden and high pharmacological treatment requirements [6, 7].

Currently available biologic therapies for severe asthma target immunoglobulin (Ig) E, interleukin (IL) -4 receptor α/IL-13, IL-5 and IL-5 receptor α [8]. However, these treatments have an indication limited to patients with: an eosinophilic phenotype; an allergic phenotype; or severe asthma with type 2 inflammation characterized by raised blood eosinophil counts and/or raised fractional exhaled nitric oxide (FeNO) levels [9–14]. Moreover, clinical studies have demonstrated that these treatments decrease exacerbation rates by approximately 50%, and that they provide variable improvements in lung function and symptom scores in some patients [15–18]. An explanation for this lack of complete efficacy may be that currently available biologics target individual cytokines or cell types, leaving other components of the asthma inflammatory response untreated. Therefore, targeting an upstream mediator of inflammation that is triggered early in the inflammatory response, and that activates a higher number of different inflammatory pathways, cytokines and cells than downstream inflammatory mediators, may have a broader effect on airway inflammation and may provide more effective asthma control. The need for such a treatment is particularly pertinent for patients with low blood eosinophil counts, including those with type 2-low inflammation, for patients whose asthma is driven by multiple inflammatory pathways, and for patients who do not respond well to treatment with existing biologic therapies.

**Fig. 1:**
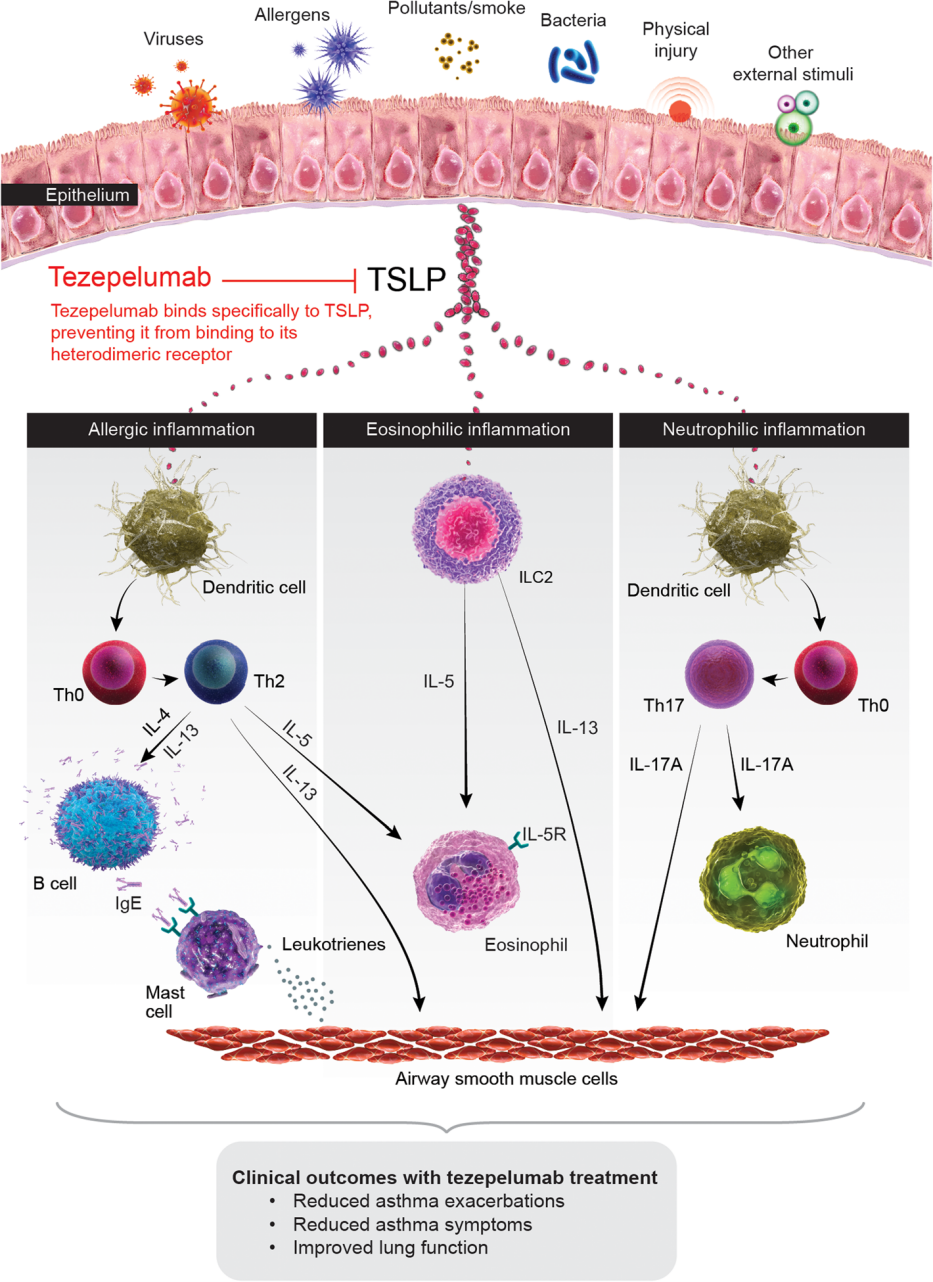
Mechanism of action by which tezepelumab improves clinical outcomes in patients with severe asthma. TSLP is released from the airway epithelium in response to insults such as viruses, allergens and pollutants, triggering an inflammatory cascade. Overexpression of TSLP can result in pathologic inflammation that can lead to asthma exacerbations, symptoms, and physiological effects such as bronchoconstriction and airway hyperresponsiveness and remodelling. Tezepelumab specifically blocks TSLP from binding to its heterodimeric receptor, thereby inhibiting the production of various inflammatory cytokines and cell types. Treatment with tezepelumab has thus far been shown to reduce eosinophils, IgE, IL-5, IL-13 and FeNO. *FeNO*, fractional exhaled nitric oxide; *IgE*, immunoglobulin E; *IL*, interleukin; *ILC2*, type 2 innate lymphoid cell; *Th*, T-helper; *TSLP*, thymic stromal lymphopoietin

Thymic stromal lymphopoietin (TSLP) is an epithelial-derived cytokine that is implicated in the initiation and persistence of airway inflammation, and is a key regulator of many downstream inflammatory pathways (Fig. 1) [19–21]. TSLP is released in response to multiple triggers associated with asthma exacerbations, including allergens, viruses and other airborne particles [19]. Expression of TSLP is increased in the airways of patients with asthma and correlates with disease severity [22, 23]. In addition to its fundamental role in asthma development, TSLP is known to be involved in the pathogenesis of other allergic conditions. Increased levels of TSLP protein are found in skin lesions of patients with atopic dermatitis [21, 24, 25] and aberrant expression of TSLP has been observed in allergic diseases of the gastrointestinal tract, including Crohn’s disease, eosinophilic oesophagitis and ulcerative colitis [26], and in cancer [27]. Higher TSLP expression has also been observed in patients with chronic obstructive pulmonary disease compared with healthy individuals [26].

Tezepelumab is a human monoclonal antibody (IgG2λ) that binds specifically to TSLP, blocking it from interacting with its heterodimeric receptor (Fig. 1) [28, 29]. In a proof-of-concept study, tezepelumab 700 mg every 4 weeks (Q4W) administered intravenously attenuated asthmatic responses to allergen challenge in patients with mild asthma [29]. In the phase 2b PATHWAY study (ClinicalTrials.gov identifier: NCT02054130), tezepelumab significantly reduced asthma exacerbations over 52 weeks by up to 71% compared with placebo in patients with severe, uncontrolled asthma, irrespective of baseline levels of inflammatory biomarkers (e.g. blood eosinophil count, FeNO and IgE), and improved lung function, asthma control and patient HRQoL [28]. In addition, tezepelumab has been shown to reduce levels of several inflammatory biomarkers, including blood eosinophils, FeNO and IgE [30]. Of currently approved biologics, only anti-IgE (omalizumab) has been shown to reduce all three of these biomarkers, although not to the same extent as tezepelumab [31]. Reductions in cytokines, including IL-5 and IL-13, have also been observed after treatment with tezepelumab [30].

**Fig. 2:**
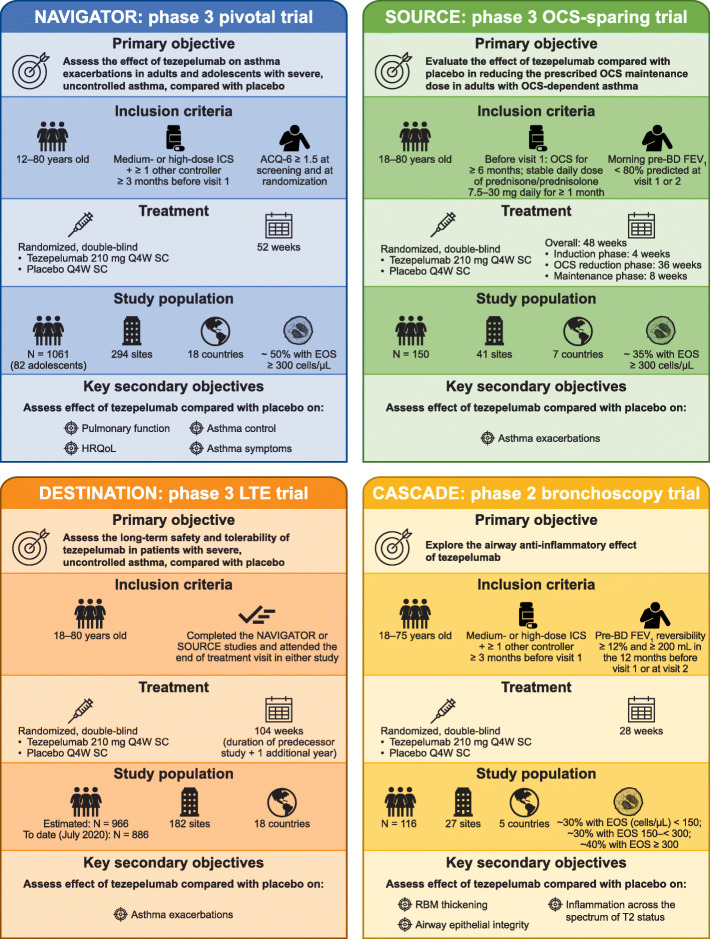
Overview of ongoing phase 2 and phase 3 clinical studies of tezepelumab. *ACQ*, Asthma Control Questionnaire; *BD*, bronchodilator; *EOS*, blood eosinophils; *FEV*_*1*_, forced expiratory volume in 1 s; *HRQoL*, health-related quality of life; *ICS*, inhaled corticosteroids; *LTE*, long-term extension; *N*, number of patients; *OCS*, oral corticosteroids; *Q4W*, every 4 weeks; *RBM*, reticular basement membrane; *SC*, subcutaneously; *T2*, type 2

Based on the reductions in exacerbations observed in a broad population of patients with severe asthma in PATHWAY, including those with low blood eosinophil counts, tezepelumab was granted breakthrough therapy designation by the US Food and Drug Administration (FDA) in 2019 for patients with severe asthma without an eosinophilic phenotype, who are receiving ICS/LABA and additional asthma controllers with or without oral corticosteroids (OCS) [32]. The efficacy and safety of tezepelumab in patients with severe, uncontrolled asthma is being investigated in a number of ongoing phase 3 trials, including NAVIGATOR (ClinicalTrials.gov identifier: NCT03347279), SOURCE (ClinicalTrials.gov identifier: NCT03406078) and DESTINATION (ClinicalTrials.gov identifier: NCT03706079) (Fig. 2). In addition, an ongoing phase 2 study (CASCADE, ClinicalTrials.gov identifier: NCT03688074) is investigating the effect of tezepelumab on airway inflammation in patients with moderate-to-severe, uncontrolled asthma. The present article describes the design and objectives of the pivotal phase 3 NAVIGATOR study, the largest study in the programme, which aims to further investigate the effect of tezepelumab on exacerbations and build on observations from the PATHWAY study, and to demonstrate the effect of tezepelumab on lung function, asthma control and HRQoL in adults and adolescents with severe, uncontrolled asthma, including those with low blood eosinophil counts.

## Methods

### Study design

NAVIGATOR is an ongoing phase 3, multicentre, randomized, double-blind, placebo-controlled, parallel group study that aims to evaluate the effect of tezepelumab 210 mg Q4W administered subcutaneously in adults (18–80 years old) and adolescents (12–17 years old) with severe, uncontrolled asthma. Patient recruitment for NAVIGATOR has been completed. A total of 1061 patients were randomized from 294 study sites in 18 countries; 82 of these patients were adolescents. Patients were stratified by region (Asia-Pacific, Central or Eastern Europe, Western Europe, Australia, North America, South America and the rest of the world) and age (adults or adolescents). To be eligible for the study, patients must have been receiving medium- to high-dose ICS (fluticasone propionate ≥500 μg dry powder formulation equivalent total daily dose) for at least 3 months before screening, and must have been taking at least one additional controller medication with or without OCS in the 3 months before the date of informed consent. Patients who had received marketed or non-investigational biologic treatments were permitted to enter the study if the last dose was taken more than 4 months or more than 5 half-lives before visit 1. Patients must also have had an Asthma Control Questionnaire (ACQ)-6 score of greater than or equal to 1.5 at both screening and randomization. Key additional inclusion and exclusion criteria are shown in Table 1.

**Table 1:** Key inclusion and exclusion criteria

Key inclusion criteria	
• Male or female, 12–80 years old, weight ≥40 kg at visit 1	
• Documented physician-diagnosed asthma for ≥12 months before visit 1, and receiving medium- or high-dose ICS (as per GINA 2017 guidelines) for 12 months before visit 1	
• Documented treatment with ICS (total daily dose corresponding to fluticasone propionate ≥500 μg dry powder formulation equivalent) plus at least one additional maintenance asthma controller medication (e.g. LABA, LTRA, theophylline) for ≥3 months before visit 1	
• Morning pre-bronchodilator FEV_1_ <80% predicted normal (<90% for patients 12–17 years old) at either visit 2 or visit 2a	
• Documented historical FEV_1_ reversibility of ≥12% and ≥200 mL in the 12 months before visit 1 OR post-bronchodilator (albuterol/salbutamol) FEV_1_ reversibility ≥12% and ≥200 mL at vist 2 or visit 2a	
• ACQ-6 score ≥1.5 at screening and at randomization	
Key exclusion criteria	
• Any clinically important pulmonary disease, other than asthma, associated with high peripheral eosinophil counts	
• Any disorder that could, in the opinion of the investigator, affect the safety of the patient or influence the study findings	
• Any clinically significant infection requiring antibiotic or antiviral treatment in the 2 weeks before visit 1 or during the run-in period	
• Helminth or parasitic infection diagnosed in the 6 months before visit 1 that has not been treated with, or is unresponsive to, standard-of-care therapy	
• History of cancer, HIV or hepatitis B or C	
• Current smokers or patients with a smoking history of ≥10 pack-years	
• Use of any marketed or investigational biologic agent in the 4 months or 5 half-lives before visit 1, or any investigational non-biologic agent in the 30 days or 5 half-lives before visit 1	
• Use of any immunosuppressive medication in the 12 weeks before randomization	
• History of anaphylaxis after biologic therapy	
• Pregnant, breastfeeding or lactating	

*ACQ-6* Asthma Control Questionnaire-6; *FEV*_*1*_ Forced expiratory volume in 1 s; *GINA* Global Initiative for Asthma; *HIV* Human immunodeficiency virus; *ICS* Inhaled corticosteroids; *LABA* Long-acting β_2_ agonist; *LTRA* Leukotriene receptor antagonist

The total study population was monitored to ensure a broad patient distribution across the following three key clinical factors. Approximately 20% of the study population comprises patients who were receiving daily medium-dose ICS (fluticasone propionate 440–500 μg dry powder formulation equivalent total daily dose) plus at least one additional controller medication, with or without OCS in the 3 months before the date of informed consent. Approximately 40% of the study population comprises patients who had at least three exacerbations in the 12 months before the date of informed consent, with the remaining 60% of patients having had two exacerbations during that time period. The study population includes similar proportions of patients with an eosinophil count of less than 300 cells/μL and patients with at least 300 cells/μL, and approximately 25% of patients have an eosinophil count of less than 150 cells/μL or of greater than 450 cells/μL. These study population targets were applied to adults only.

**Fig. 3:**
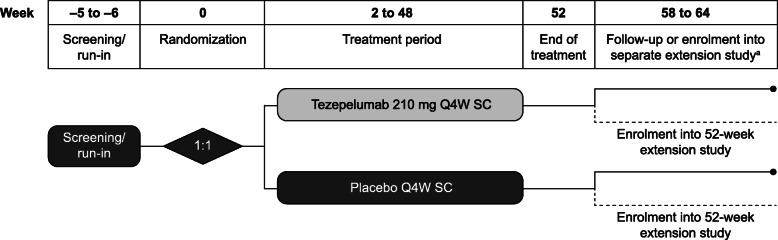
Study design. ^a^Patients who enrol in the extension study on the same day as the end-of-treatment visit in NAVIGATOR will not attend follow-up visits at week 58 and week 64. *Q4W*, every 4 weeks; *SC*, subcutaneously

The study comprises a 5- to 6-week screening and run-in period, followed by a treatment period of 52 weeks and a post-treatment follow-up period of 12 weeks (Fig. 3). Patients were randomized in a 1:1 ratio to receive tezepelumab 210 mg Q4W subcutaneously (administered using a single-use vial and syringe) or placebo Q4W for 48 weeks. Neither treatment will be administered at week 52. For the duration of the study, all patients will receive their prescribed ICS plus additional controller medications without change. Patients will be permitted to use short-acting β_2_ agonists as relief medication for symptoms of asthma as required. Patients who complete the treatment period as defined in the study protocol may be eligible to enrol in a separate extension study (DESTINATION); these patients will not attend follow-up visits at weeks 58 and 64. Patients who discontinue treatment early will be encouraged to undergo applicable study-related visits and/or procedures for the 52-week study period.

Owing to the COVID-19 virus pandemic, the NAVIGATOR protocol was amended. This amendment was necessary to address the issue of social distancing and the possibility that site visits would be limited. If site visits by patients were not possible, efficacy (e.g., exacerbations, spirometry and patient-reported outcomes) and safety data may not have been collected. In addition, patients would not have been able to receive study drug. The amendment specifically allowed for virtual visits (instead of site visits) to collect appropriate efficacy and safety information (except for spirometry), and for at-home dosing of study drug when possible. Notification of the planned changes to the study protocol as a result of the pandemic was provided to each study site, and virtual visits and at-home dosing commenced, if permitted by the local regulatory authorities, ethics committees and Institutional Review Boards, and if required. Instructions for at-home dosing of study drug by a healthcare professional (e.g. transportation, preparation, handling and administration) were provided to each site. To date (July 2020), only 17 of over 17,000 completed visits have taken place virtually, and only 25 of approximately 13,000 doses were missed by patients who have completed the study or who are still participating in the study, owing to travel restrictions or to patient choice. In addition, no at-home dosing has taken place.

Written informed consent was obtained from all patients or their guardians before enrolment into the study. The study is being conducted in accordance with the principles established in the Declaration of Helsinki and the International Council for Harmonisation guidelines for good clinical practice.

### Objectives and outcome measures

A summary of the primary and secondary objectives and endpoints for this study is given in Table 2. The primary objective of this study is to assess the effect of tezepelumab compared with placebo on asthma exacerbations over the 52-week treatment period. This will be assessed by the annualized asthma exacerbation rate (AAER). In addition to the overall population, the primary objective will be assessed in a subgroup of patients with blood eosinophil counts less than 300 cells/μL.

**Table 2:** Primary and secondary objectives and endpoints

Objective	Endpoint(s)
*Primary objective*	
Assess the effect of tezepelumab on asthma exacerbations in adults and adolescents with severe, uncontrolled asthma, compared with placebo	AAER
*Key secondary objectives*	
Assess the effect of tezepelumab on pulmonary function compared with placebo	Change from baseline in pre-bronchodilator FEV_1_
Assess the effect of tezepelumab on HRQoL compared with placebo	Change from baseline in AQLQ(S) + 12 total score
Assess the effect of tezepelumab on asthma control compared with placebo	Change from baseline in ACQ-6 score
Assess the effect of tezepelumab on asthma symptoms compared with placebo	Change from baseline in weekly mean daily Asthma Symptom Diary score
*Other secondary objectives*	
Assess the effect of tezepelumab on other endpoints associated with exacerbations	Time to first asthma exacerbation Proportion of patients with ≥1 exacerbation Annualized rate of exacerbations associated with hospital, ER or urgent care visits
Assess the effect of tezepelumab on biomarkers	Change from baseline in FeNO, peripheral blood eosinophil count and total serum IgE
Assess the effect of tezepelumab on other asthma control metrics	Weekly mean rescue medication use Weekly mean morning and evening PEF Weekly mean number of night-time awakenings
Assess the effect of tezepelumab on healthcare resource use and productivity loss due to asthma	Asthma-specific resource use: for example, unscheduled physician visits, use of other asthma medications WPAI + CIQ score
Evaluate the pharmacokinetics and immunogenicity of tezepelumab	Pharmacokinetics: serum trough concentrations Immunogenicity: incidence of anti-drug antibodies
Assess the effect of tezepelumab on general health-related quality of life	EQ-5D-5L
Assess the effect of tezepelumab on patient and clinical impression of overall asthma severity	PGI-C/S CGI-C

All objectives relate to tezepelumab 210 mg Q4W SC

*AAER* Annualized asthma exacerbation rate; *ACQ-6* Asthma Control Questionnaire-6; *AQLQ(S) + 12* Asthma Quality of Life Questionnaire standardized for patients 12 years and older; *CGI-C* Clinician Global Impression of Change; *EQ-5D-5L* European Quality of Life – 5 Dimensions 5 Levels; *ER* Emergency room; *FeNO* Fractional exhaled nitric oxide; *FEV*_*1*_ Forced expiratory volume in 1 s; *IgE* Immunoglobulin E; *PEF* Peak expiratory flow; *PGIC/S* Patient Global Impression of Change/Severity; *Q4W* Every 4 weeks; *SC* Subcutaneous; *WPAI + CIQ* Work Productivity and Activity Impairment Questionnaire and Classroom Impairment Questionnaire

Key secondary objectives include assessing the effect of tezepelumab compared with placebo on pulmonary function (pre-bronchodilator forced expiratory volume in 1 s [FEV_1_]) and on patient-reported outcomes, including HRQoL (using the Asthma Quality of Life Questionnaire standardized for patients 12 years and older [AQLQ(S) + 12]), asthma control (using the ACQ-6) and asthma symptoms (using the Asthma Symptom Diary, which is underdoing validation in line with FDA requirements for patient-reported outcome measures [33–35]). Additional secondary objectives include assessing the effect of tezepelumab on type 2 inflammatory biomarkers (including FeNO, blood eosinophils and total serum IgE), as well as on other endpoints related to exacerbations (including time to first exacerbation, the proportion of patients without an exacerbation, and the annualized rate of exacerbations associated with hospital, emergency room or urgent care visits), asthma control measures (including rescue medication use, night-time awakenings and peak expiratory flow) and healthcare resource use.

In addition, NAVIGATOR will investigate a number of exploratory outcomes including, but not limited to, the effect of tezepelumab on: patient health status (using St George’s Respiratory Questionnaire [SGRQ]); disease activity in a subset of adult patients (using daily domiciliary FeNO measurements); and sino-nasal specific HRQoL (using the Sino-Nasal Outcome Test [SNOT]-22) in patients with a history of nasal polyps.

During the study, the safety and tolerability of tezepelumab will be evaluated by monitoring adverse events; serious adverse events; vital signs; clinical chemistry, haematology and urinalysis parameters; and digital electrocardiograms. A data safety monitoring board (an expert advisory group that functions independently from all other study personnel) will monitor safety aspects related to the involvement of adolescent patients in the study, and will review safety data for adults to provide context for what is observed in adolescents. The data safety monitoring board will evaluate cumulative safety and other clinical trial data at regular intervals, and will make appropriate recommendations based on the available data. Serum samples will be collected throughout the study for analysis of tezepelumab pharmacokinetics (serum concentrations) and analysis of the potential immunogenicity of tezepelumab, as measured by the incidence of anti-drug antibodies and characterization of their neutralizing potential.

### Statistical considerations

All statistical analyses of primary and secondary efficacy outcomes will be performed after the primary database lock, which will be conducted after the last patient completes week 52 of the study. A second database lock will be conducted when all patients have completed their follow-up visits (or entered the long-term extension study).

Statistical analyses of efficacy outcomes will be performed on all patients randomized to study treatment who receive at least one dose of tezepelumab or placebo, according to randomized treatment assignment (full analysis set). Statistical analysis of safety and anti-drug antibody incidences will be performed on the safety analysis set (all patients who receive at least one dose of tezepelumab or placebo), according to treatment received. Pharmacokinetic analyses will be performed on all patients in the full analysis set who receive tezepelumab; samples assumed to be affected by factors such as specific important protocol deviations (e.g. use of non-permitted medications or receipt of incorrect study medication) will not be included.

AAER over 52 weeks (primary efficacy endpoint) will be analysed using a negative binomial regression model, with the total number of asthma exacerbations experienced by a patient over the 52-week study period used as a response variable. Treatment, region, age (adolescents or adults) and history of exacerbations (two or more than two in the previous 12 months) will be included as covariates in the model. The logarithm of the time at risk for an exacerbation during the study (excluding the duration of an exacerbation and the 7-day period after an exacerbation, during which recurrence of symptoms will be considered part of the initial exacerbation and not a new exacerbation) will be used as an offset variable in the model. Exacerbations occurring more than once in a 7-day period will be considered the same exacerbation in the analysis. Patients lost to follow-up and those who withdraw consent will be the only sources of missing data for this analysis. Missing data from study discontinuation will be modelled based on what is observed during the study using direct likelihood approaches (which assumes data are missing at random), and sensitivity analyses using multiple imputation will be performed to explore the effect of missing data on the reliability of the results. A similar model will be used for subgroup analyses (including patients with blood eosinophil counts less than 300 cells/μL), with additional factors for the relevant subgroup variable and its interaction with treatment.

Changes from baseline for key secondary endpoints in the tezepelumab group will be compared with those seen in the placebo group using a mixed model for repeated measures, which will estimate the effect of treatment at week 52 and its 95% confidence interval for each endpoint. The response variable in the model will be the change from baseline at each scheduled post-randomization visit up to and including week 52, irrespective of whether the patient remained on treatment and/or took other treatments. Treatment, visit, region, age and treatment-by-visit interaction will be included as factors in this model. The baseline value of the corresponding endpoint will also be included in the model as a continuous linear covariate. As for the analysis of the primary endpoint, missing data will be modelled based on what is observed during the study using direct likelihood approaches. Any additional statistical analyses required due to COVID-19 will be pre-specified in the statistical analyses plan before sponsor unblinding and will be described when the results for this study are reported.

**Fig. 4:**
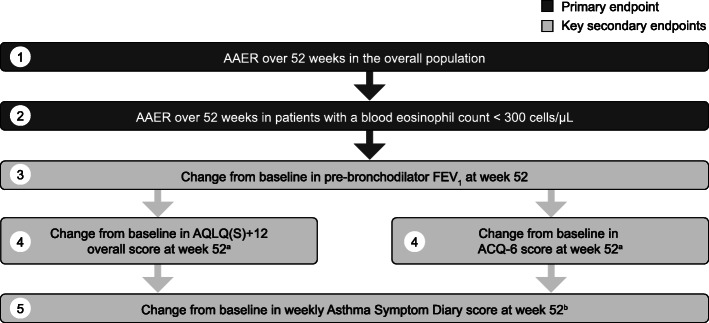
Hierarchical testing strategy for primary and key secondary endpoints. Testing will be performed at a two-sided 5% significance level unless otherwise stated. ^a^A truncated Hochberg adjustment will be used for level 4. As such, the higher of the two level 4 *p* values will be evaluated at a 3.75% significance level (two-sided). If it is significant at the 3.75% level, both level 4 null hypotheses will be rejected, and testing will proceed to level 5. If it is not significant at the 3.75% level, the lower of the two level 4 *p* values will be evaluated at a 2.5% significance level (two-sided). If significant, the relevant null hypothesis will be rejected, and testing will proceed to level 5. ^b^If both null hypotheses are rejected at level 4, level 5 will be tested at a two-sided 5% significance level. If only one null hypothesis is rejected at level 4, level 5 will be tested at a two-sided 1.25% significance level. *AAER*, annualized asthma exacerbation rate; *ACQ-6*, Asthma Control Questionnaire-6; *AQLQ(S) + 12*, Asthma Quality of Life Questionnaire standardized for patients 12 years and older; *FEV*_*1*_, forced expiratory volume in 1 s

The overall type 1 error rate will be strongly controlled at the 0.05 level across the primary and key secondary endpoints. To account for multiplicity, a hierarchical testing strategy will be used to test the primary endpoint and then the key secondary endpoints (Fig. 4). To assess the primary efficacy objective across a broad population of patients, the subgroup of patients with a baseline eosinophil count less than 300 cells/μL was added to the multiple testing procedure after the primary endpoint and before the key secondary endpoints. With approximately 530 patients per treatment group it is estimated that, using the level of error control described, the power for the primary and key secondary endpoints will be at least 90%. For the primary endpoint (in all patients), assuming a placebo exacerbation rate of 0.9, a shape parameter of 2.4 (over-dispersion) and a dropout rate of 10% (uniform over the study), there will be > 99% power to detect an AAER reduction of 50% at a two-sided significance level of 1%. For AAER in patients with a blood eosinophil count less than 300 cells/μL, assuming a placebo rate of 0.6 and that half of the overall patient population will be in this group (i.e. 265 patients per treatment group), there will be 94% power to detect a rate reduction of 50% at a two-sided significance of 5% (assuming the same shape parameter and dropout assumptions as those for the overall population). For each of the key secondary endpoints (to be assessed in all patients), change from baseline in FEV_1_, AQLQ(S) + 12 overall score, ACQ-6 score and Asthma Symptom Diary score, the nominal power will be 95% or higher, assuming standard deviations of 400 mL and 1.3, 1.3 and 1.3 units, respectively, and true differences of 100 mL and 0.3, 0.3 and 0.3 units, respectively. The minimum detectable differences being significant under these assumptions are 50 mL and 0.16, 0.16 and 0.16 units, respectively.

## Discussion

Tezepelumab is a human monoclonal antibody that blocks TSLP from interacting with its heterodimeric receptor, resulting in the inhibition of multiple downstream inflammatory pathways [28–30]. The phase 2b PATHWAY study demonstrated that blocking TSLP with tezepelumab may be an effective strategy for the treatment of severe, uncontrolled asthma, with reductions in exacerbations observed irrespective of baseline inflammation status [28, 36]. In clinical studies to date, which have included healthy individuals, patients with asthma and patients with atopic dermatitis, tezepelumab has been well tolerated with no safety signals identified [28, 29, 37–39].

NAVIGATOR aims to further investigate the effect of tezepelumab on exacerbations and build on findings from the PATHWAY study, and to evaluate the effect of tezepelumab on lung function, asthma control and HRQoL in adults and adolescents with severe, uncontrolled asthma, who were receiving medium- to high-dose ICS plus at least one additional controller medication with or without OCS, in addition to evaluating its safety in this patient population. NAVIGATOR will also evaluate the effect of treatment with tezepelumab in patients without an eosinophilic phenotype. The target number of patients for NAVIGATOR (*N* = 1060) was achieved, making it the largest study in the tezepelumab phase 3 programme and the largest clinical study of tezepelumab in severe asthma to date. This pivotal study will allow the benefit–risk profile of tezepelumab in the treatment of asthma to be characterized further, and it will provide greater understanding of which patients benefit most from treatment with tezepelumab. The primary (AAER) and key secondary (lung function, asthma control and HRQoL) endpoints of NAVIGATOR are well-accepted efficacy measures for studies of patients with severe asthma. In the phase 2b PATHWAY study, these endpoints clearly differentiated the benefits of tezepelumab from those of placebo [28].

A phase 3 OCS-sparing study, SOURCE, is running in parallel with the NAVIGATOR study and has a primary objective of assessing the effect of tezepelumab compared with placebo in reducing the prescribed maintenance OCS dose over 48 weeks in adults with OCS-dependent asthma. SOURCE aims to demonstrate that treatment with tezepelumab in patients with severe asthma is associated with reductions in exacerbation rates and improvements in lung function and HRQoL, while reducing OCS dose. Patients who complete the NAVIGATOR or SOURCE studies have the opportunity to enrol in DESTINATION, a phase 3 long-term extension study aiming to evaluate the safety and efficacy of tezepelumab over a period of up to 2 years (inclusive of the treatment period of either predecessor study). The primary objective of DESTINATION is to assess the long-term safety and tolerability of tezepelumab compared with placebo. The clinical effect of tezepelumab after treatment cessation will also be investigated during DESTINATION, and the study aims to elucidate the potential long-term treatment benefits of tezepelumab in patients with severe, uncontrolled asthma. Together, this set of phase 3 clinical studies aims to provide efficacy and safety data in a large population of patients with severe asthma, including those with different asthma phenotypes. Also running in parallel with these studies is CASCADE, a phase 2 bronchoscopy study that aims to investigate the mechanisms by which tezepelumab confers clinical benefit to patients with moderate to severe, uncontrolled asthma. The primary objective of CASCADE is to explore the airway anti-inflammatory effect of tezepelumab by investigating changes in levels of inflammatory cells present in bronchoscopic biopsies over 28 weeks.

Given the upstream location of TSLP in the inflammatory cascade and its role in the initiation of the inflammatory response [19–21], its blockade is anticipated to have a broad impact on the spectrum of inflammatory responses seen in patients with severe asthma. Therefore, it is expected that patients with severe asthma may benefit from treatment with tezepelumab, irrespective of their disease phenotype. This hypothesis is supported by results from the PATHWAY study, which demonstrated that tezepelumab reduced exacerbations in patients with severe, uncontrolled asthma, irrespective of their baseline inflammation status [28, 36]. In a subgroup analysis of the PATHWAY population, reductions in exacerbations were observed in patients with a blood eosinophil count of less than 300 cells/μL [28, 36]. NAVIGATOR aims to further assess the efficacy of tezepelumab across a range of severe asthma phenotypes, including patients without an eosinophilic phenotype. To ensure that patients with a broad range of different severe asthma phenotypes were enrolled in NAVIGATOR (e.g. high and low eosinophil counts, medium- and high-dose ICS users and different numbers of exacerbations in the previous year), patient entry in the study was monitored for these subgroups.

Selection of a 210 mg Q4W dose regimen for phase 3 studies was based on efficacy data and an exposure–response analysis from the phase 2b PATHWAY study [40]. AAER data from PATHWAY indicated that the 210 mg Q4W regimen provided improved efficacy over the 70 mg Q4W regimen, whereas the 280 mg every 2 weeks regimen did not further reduce AAER [28]. Furthermore, an exposure–response relationship against the AAER and the pharmacodynamic endpoint, FeNO, was identified from an analysis of data from PATHWAY [40], which also demonstrated that the 210 mg Q4W dose provided similar pharmacodynamic effects to the 280 mg Q2W dose.

Key learnings from the phase 2b study that have affected the design of NAVIGATOR are primarily related to ensuring the appropriate patient population was enrolled. To ensure that patients were symptomatic at randomization, an ACQ-6 score of greater than or equal to 1.5 was required both at screening and randomization in NAVIGATOR; this score was only required on one of these occasions during PATHWAY. Additionally, NAVIGATOR inclusion criteria were designed so that enrolled patients must have experienced two or more exacerbations in the previous year, whereas patients in PATHWAY could have experienced at least two exacerbations in the previous year, or at least one exacerbation leading to hospitalization.

Additional secondary endpoints (e.g. healthcare resource use and Asthma Symptom Diary score) and exploratory endpoints (effects on nasal polyposis symptoms in adults, daily domiciliary FeNO and SGRQ score) are included in NAVIGATOR based on a greater understanding of the potential effects of tezepelumab following the phase 2b study. The Asthma Symptom Diary will be used to assess the severity of asthma symptoms, nocturnal awakenings and activity limitations in study participants, and has been created in line with FDA recommendations that patient-reported outcome measures are developed based on qualitative information and have evidence supporting reliability, validity and responsiveness [33–35]. As such, the symptom content of the Asthma Symptom Diary is based on concept elicitation and cognitive interviews in patients with moderate-to-severe, persistent asthma, and previous studies have supported the content validity and psychometric characteristics [33–35]. The SGRQ is included to assess the health status of patients in addition to the AQLQ(S) + 12. The SGRQ comprises a greater number of items relating to respiratory symptoms, daily activities and psychosocial/emotional impacts than the AQLQ(S) + 12, and it covers a broader range of disturbances that patients with severe asthma may experience [41]. In addition, the SNOT-22 questionnaire will be used to assess health-related outcomes in a subset of adult patients with nasal polyposis, which will provide data on the potential effect of tezepelumab in patients with this comorbid inflammatory condition, which is present in up to 40% of patients with asthma [42].

Owing to the COVID-19 pandemic, the NAVIGATOR protocol was amended to enable social distancing and address the possibility that site visits would be limited. It was possible to implement virtual visits and at-home dosing rapidly, which was important in order to reduce the risk of exposing study participants to SARS-CoV-2. To date (July 2020), as a result of the changes implemented, a very small proportion of visits (17 out of over 17,000) have taken place virtually, and a very small number of doses (25 out of approximately 13,000) have been missed. In addition, no at-home dosing has taken place. Given that enrolment for NAVIGATOR was complete and that a large proportion of the study data had been collected before COVID-19 became a global pandemic, the impact on this clinical study to date is considered to be low.

## Conclusion

Patients with severe, uncontrolled asthma have a significant unmet need for new treatments that have broader effects on airway inflammation, and that provide greater improvements in asthma outcomes than currently approved biologics and standard-of-care therapies. NAVIGATOR is evaluating the effect of tezepelumab in this patient population, and has enrolled patients with a broad range of asthma phenotypes at baseline, including those with low blood eosinophil counts. It is the largest clinical study of tezepelumab in severe, uncontrolled asthma to date. NAVIGATOR aims to provide further evidence of the findings from earlier clinical studies with tezepelumab, and to demonstrate further its potential to provide improvements in lung function, asthma control and HRQoL to patients with severe, uncontrolled asthma.

## Data Availability

Not applicable.

## Abbreviations

AAER: Annualized asthma exacerbation rate
ACQ-6: Asthma control questionnaire-6
AQLQ(S) + 12: Asthma quality of life questionnaire standardized for patients 12 years and older
FDA: Food and drug agency
FeNO: Fractional exhaled nitric oxide
FEV_1_: Forced expiratory volume in 1 s
HRQoL: Health-related quality of life
ICS: Inhaled corticosteroids
Ig: Immunoglobulin
IL: Interleukin
LABAs: Long-acting β_2_-agonists
OCS: Oral corticosteroids
Q4W: Every 4 weeks
SGRQ: St George’s respiratory questionnaire
SNOT-22: Sino-nasal outcome test-22
TSLP: Thymic stromal lymphopoietin
